# Longitudinal Analysis of Adolescent Adjustment: The Role of Attachment and Emotional Competence

**DOI:** 10.3390/children9111711

**Published:** 2022-11-08

**Authors:** Tamara Jiménez-Rodríguez, Usue De la Barrera, Konstanze Schoeps, Selene Valero-Moreno, Inmaculada Montoya-Castilla

**Affiliations:** 1 Department Personality, Assessment and Psychological Treatments, Faculty of Psychology and Speech Therapy, Universitat de Valencia, Blasco Ibáñez, 21, 46010 Valencia, Spain; 2 Department Developmental and Educational Psychology, Faculty of Psychology and Speech Therapy, Universitat de Valencia, Blasco Ibáñez, 21, 46010 Valencia, Spain

**Keywords:** adolescence, adjustment, attachment, emotional competence, SEM, fsQCA models

## Abstract

Emotional competencies as well as secure attachment relationships with peers are protective factors that facilitate psychological adjustment among adolescents. In this study, we will analyse how these socio-emotional factors influence adolescents’ emotional symptoms, conduct problems and peer problems. The participants were 815 Spanish adolescents aged 12–17 years (*M* = 13.69; *SD* = 1.21) who completed the Strengths and Difficulties Questionnaire (SDQ), the Parent and Peer Attachment Inventory (IPPA) and the Emotional Skills and Competence Questionnaire (ESCQ). Besides descriptive analyses, such as Pearson’s bivariate correlations, two different methodologies were combined to predict adolescent adjustment: structural equation modelling (SEM) and fuzzy set qualitative comparative analysis (fsQCA). The results show that secure attachment with peers and high emotional competence predict better psychological adjustment (low levels of emotional symptoms, conduct problems and peer problems), while insecure peer attachment and low emotional competence predict maladjustment. These results emphasise the role of socio-emotional variables in the promotion of psychological adjustment in adolescence through the implementation of emotional education programs.

## 1. Introduction

Adolescence is a developmental stage where physical, neurocognitive, social, affective and academic changes occur and is considered a period of significant vulnerability [1]. During this stage, adolescents build their identity and are at greater risk of suffering emotional and behavioural problems [2,3] and being exposed to social stressors [4,5]. In this sense, adolescence is a lifecycle stage with an increased likelihood of developing psychological and adjustment problems [6].

### 1.1. Psychological Adjustment

Psychological adjustment is the ability to adapt appropriately to the environment, addressing emotional, behavioural and social aspects [7,8]. Good adjustment is associated with coping with life challenges and responding appropriately to environmental demands [9]. Adolescents with better adjustment engage in more positive social behaviours [10,11].

It has been observed that a high percentage of adolescents will experience psychological adjustment difficulties throughout their lives [12]. These adolescents who present emotional and behavioural difficulties will be more likely to develop mental disorders during their adulthood [13].

Family conflicts or lack of communication with caregivers are related to greater emotional and behavioural problems [14] and, accordingly, to poorer psychological adjustment [11,15]. However, secure attachment, cohesion and family support facilitate adjustment and positive peer relationships [16,17]. Additionally, having a healthy friendship network during adolescence contributes to developing skills, emotional stability and adjustment [11,18].

Studies report that emotional, behavioural and peer relationship problems are common during adolescence [19,20,21]. These problems include affective difficulties that involve somatic complaints, anxiety, nervousness in new situations, lack of self-confidence, feelings of sadness and fear, behavioural difficulties that involve perceived loss of control, lack of obedience, frequent fighting, manipulating others, lying and stealing, and social difficulties that involve feelings of loneliness, playing alone, not having friends, feeling teased by others, having better relationships with adults than with peers and being disliked by peers [19]. These problems influence the psychological functioning of adolescents, as well as their personal, academic, family and social lives [22].

### 1.2. Peer Attachment

Attachment theory explains the relationships between children and their caregivers during early childhood [23]. How these attachments interact is essential in the formation process throughout childhood and into adolescence, showing that those adolescents who feel close to their caregivers have a better emotional and social development [14].

In adolescence, caregivers remain a source of support and protection; however, peers gradually become solid socialising agents [24,25,26]. Although attachment develops during the early years of life, it continues to influence adolescents’ psychological adjustment [27,28]. Therefore, it is essential to understand how adolescents relate to their peers and how this attachment affects their emotions and behaviours [29].

Secure peer attachment is a relationship based on mutual understanding, trust and the belief that the other person will take one’s needs and concerns into account, understand and respond when the other person communicates their feelings [30,31]. On the contrary, adolescents with insecure attachment experience more feelings of alienation and tend to isolate themselves from their peers [5,32].

Peer relationships become social, emotional and behavioural predictors during adolescence [5]. Adolescents who share interests and concerns with their peers and feel their opinions and feelings are accepted have fewer emotional symptoms and behavioural problems and, conversely, those who are distant, confrontational, have poor communication, lack trust and isolate themselves from their peer group tend to experience more emotional symptoms and behavioural problems [32,33,34,35,36]. Peer attachment is negatively associated with emotional and behavioural difficulties longitudinally [37,38].

In terms of attachment dimensions, emotional symptoms and behavioural problems are negatively related to peer trust and communication and positively related to alienation [5,32,33,36].

### 1.3. Emotional Competencies

During adolescence, it is important to identify and address those factors that influence adolescents’ adjustment and promote their strengths, with emotional competencies being one of the most influential constructs [3,39].

Emotional competencies are a set of skills related to emotions [40,41]. This construct derives from the emotional intelligence model proposed by Mayer and Salovey [42], which assesses emotional intelligence as a skill and is comprised of four dimensions: (a) emotional perception and expression, (b) use of emotion to facilitate thinking, (c) understanding of emotion and (d) emotional regulation [43].

Emotional competencies are developed and trained during childhood and adolescence, enabling previous experiences to guide future behaviour [44]. These skills help adolescents identify and become aware of their feelings and regulate their emotional reactions towards themselves and others [43].

Research suggests that emotional skills are related to psychological adjustment [45,46,47,48] and help in predicting it [49,50].

In this sense, those adolescents who present higher levels of emotional skills experience fewer behavioural and emotional problems [3,47,48,51]. In particular, emotional perception is found to positively predict emotional problems and influence behavioural and expression problems, and emotion management is negatively related to emotional symptoms and behavioural problems [3,51]. Additionally, adolescents who pay too much attention to their emotions while lacking adequate emotional expression and skills to help regulate them could negatively affect their psychological adjustment [3,52]. Thus, high levels of perceiving, understanding, expressing and managing emotions are related to low levels of emotional symptoms [53].

Emotions play a determining role in social interactions, meaning that the ability to regulate one’s own emotions becomes a crucial skill for establishing and maintaining interpersonal relationships [54]. In this sense, emotional competencies influence peer relationships [55]. Thus, adolescents with higher emotional competence develop better interpersonal and social skills, have more friends and establish healthier and positive social relationships [56,57,58]. In contrast, adolescents with poor emotional skills have fewer resources to deal with interpersonal conflicts and use aggression to solve problems [59,60].

### 1.4. The Current Study

Previous studies highlight that peer attachment and emotional competencies are related to adolescent psychological adjustment [27,38,48,50]. However, few studies on the adolescent population examine long-term adjustment outcomes through longitudinal designs [46]. Therefore, more longitudinal studies are needed to help confirm the causal relationships of psychological adjustment with different variables and determine whether psychological adjustment prediction remains stable over time. Furthermore, two different but complementary methods for predicting adolescent adjustment are combined in this study to achieve a broader understanding of this phenomenon. A predictive structural equation model (SEM) was conducted to estimate latent variables (constructs) from observed variables (items), while accounting for measurement error in the sample data. In addition, fuzzy set qualitative comparative analysis (fsQCA) was used to explore a variety of different paths that combine multiple predictors in different ways.

This study aimed to examine how peer attachment and emotional competencies impact psychological adjustment in adolescence over time. Considering previous research, the following hypotheses have been proposed: (1) secure peer attachment (high trust, high communication and low alienation) and optimal emotional competence (low emotional perception, high emotional expression and high emotional management) will be negatively associated with emotional symptoms, (2) secure peer attachment (high trust, high communication and low alienation) and optimal emotional competence (low emotional perception, high emotional expression and high emotional management) will be negatively associated with conduct problems and (3) secure peer attachment (high trust, high communication and low alienation) and optimal emotional competence (low emotional perception, high emotional expression and high emotional management) will be negatively associated with peer problems.

## 2. Materials and Methods

### 2.1. Participants

The participants in this study were 815 adolescents aged 12–17 years (*M* = 13.69; *SD* = 1.21). The distribution according to sex was 56.5% female and 43.5% male, and according to age, 21.9% were 12 years old, 21.3% were 13 years old, 28.4% were 14 years old, 22.9% were 15 years old, 5.1% were 16 years old, and 0.5% were 17 years old. They were all studying between the first and fourth year of Compulsory Secondary Education (ESO) in different educational centres in the Valencian Community. The sample was collected at two different time points over six months, and only those adolescents who participated in both assessments were included.

### 2.2. Procedure

This study is a two-stage longitudinal design with six months between the two assessments. All variables were measured at the first time point (T1, January 2016) and at the second time point (T2, June 2017).

To conduct this study, an assessment booklet for adolescents was designed, which received the approval of the Regional Ministry of Education of the Valencian Community. The study was also accepted by the ethics committee of the research institution, in compliance with ethical values and in accordance with the principles of the Declaration of Helsinki (World Medical Association, 2013). Subsequently, different educational centres in the Valencian Community were contacted, and upon their acceptance, the parents of the participating students were informed about the aim of the study. Parents gave their informed consent and the adolescents later completed the questionnaires individually during school hours in the presence of the evaluating psychologist and the teacher responsible for the group. The assessment lasted approximately 50 min and was carried out collectively. This assessment was repeated six months later under the same conditions described above.

### 2.3. Measures

#### 2.3.1. Psychological Adjustment

The psychological adjustment was assessed using the Strengths and Difficulties Questionnaire [61] in its Spanish version [62]. Social-emotional and behavioural circumstances are assessed with the instrument. It is composed of 25 items, divided into 5 different 5-item subscales: emotional symptoms, behavioural problems, peer problems, hyperactivity and prosocial behaviour. A 4-point Likert scale was used to answer each item (0 = strongly disagree, 4 = strongly agree). Only the emotional symptoms, behavioural problems and peer problems scales were used for this study. Reliability for the sample was adequate for emotional symptoms, α = 0.67, behavioural problems, α = 0.54, and peer problems, α = 0.32.

#### 2.3.2. Peer Attachment

Peer attachment was assessed with the Parent and Peer Attachment Inventory [63] in its validated Spanish version [64]. This instrument assesses adolescents’ perception of their relationship with their mothers, fathers and peers. The questionnaire consists of 75 items divided into 3 subscales of 25 items each, and the total score is evaluated on a 5-point Likert-type scale (1 = never or rarely true, 5 = always or usually true). It provides differentiated scores for father, mother and peers on three dimensions: trust, communication and alienation. For this study, only the peer attachment scale was considered. Reliability for the sample was adequate: trust, α = 0.85, communication, α = 0.84, and alienation, α = 0.62.

#### 2.3.3. Emotional Competencies

Emotional competencies were assessed through the Emotional Skills and Competence Questionnaire [65] adapted to Spanish [66]. This questionnaire consists of 21 items, divided into 3 dimensions: emotional perception (7 items), emotional expression (7 items) and emotional management (7 items). A 6-point Likert scale was used to answer each item (1 = never, 6 = always). The reliability for the three scales was adequate: perception and understanding, α = 0.85, expression and labelling, α = 0.89, and management and regulation, α = 0.79.

### 2.4. Analysis Plan

First, descriptive analyses and bivariate correlations (Pearson’s correlation coefficient) were carried out using SPSS (Version 24). Afterwards, Mplus (Version 8.3) was used to run a predictive structural equation model (SEM). The model analysed the direct effects of peer attachment and emotional competencies (independent variables) on emotional symptoms, conduct problems and peer relationship problems (dependent variables). Considering the possible non-normality of the data, robust maximum likelihood (ML) estimation was used. As recommended by Hu and Bentler [67], the following models’ goodness-of-fit statistics and indices were used: chi-square statistic (χ^2^), the comparative fit index (CFI), the root mean square error of approximation (RMSEA) and the standardised root mean square residual (SRMR). For the interpretation of the models’ goodness-of-fit indices, values below 0.06 are recommended for the RMSEA and the SRMR [67] and values above 0.90 for the CFI [68].

Second, to perform fuzzy set qualitative comparative analysis (fsQCA) models, the values were calibrated and transformed into fuzzy set responses. Participants with missing values were previously excluded from the analysis. The following analyses were conducted using the Fs/QCA software (Version 3.0) (University of California, Irvine, CA, Orange County, USA). First, items of dimensions were multiplied to build the conditions (variables), as previous studies suggested [69,70]. Percentiles were calculated to determine three thresholds [71]. Specifically, the 90th percentile is high or fully in-set agreement, while the 10th percentile indicates low or fully out-of-set agreement. The intermediate level of agreement (neither within nor outside the set) is the 50th percentile. The values of the conditions (variables) must range between 0 and 1 and were calculated considering the three thresholds. After calibration and transformation, necessary and sufficient analyses were performed. A condition is necessary when it must always be present for a given outcome to occur, while a condition is sufficient when it leads to a given outcome but can also be achieved by other conditions or combinations [72]. In necessary analysis, a condition is necessary if its consistency is above 0.90. In sufficient analysis, the fuzzy set membership scores are transformed into a truth table by a truth table algorithm. The truth table indicates all logically possible combinations of causal conditions and provides the empirical outcome of each configuration [73]. The cut-off point from which each condition of the combination is considered reliable is 0.70 [72]. The analyses provide three solutions (complex, intermediate and parsimonious). It is recommended to pay attention to the intermediate solution [72].

## 3. Results

### 3.1. Preliminary Analyses

Descriptive statistics and Pearson’s bivariate correlations are presented in Table 1.

Preliminary correlations revealed that peer attachment and emotional competencies were significantly correlated at T1. The correlations also indicated that all three dimensions of peer attachment at T1 were significantly related to adolescents’ adjustment at T2. Correlations also showed significant associations between the three emotional abilities at T1 with emotional symptoms and conduct problems at T2, but the link with peer problems at T2 was only significant with emotional management at T1.

### 3.2. Structural Equation Model (SEM)

**Figure 1:**
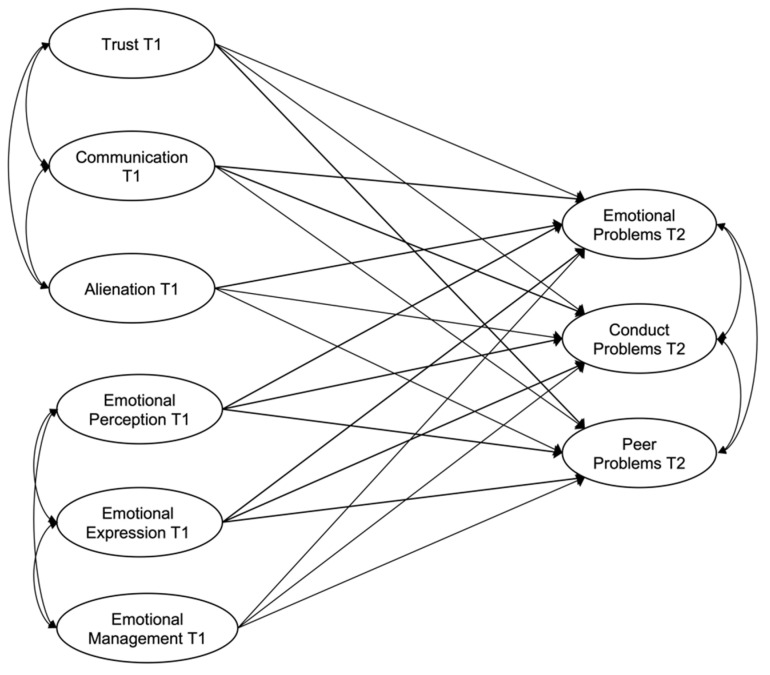
Initial model to predict emotional symptoms, conduct problems and peer problems.

First, a fully structural (theoretical) model was proposed. This model aimed to test whether a series of independent variables—peer attachment (trust, communication and alienation) and emotional competence (perceiving, expressing and managing emotions)—affect a series of dependent variables—emotional symptoms, conduct problems and peer problems. This first model is shown in Figure 1.

**Figure 2:**
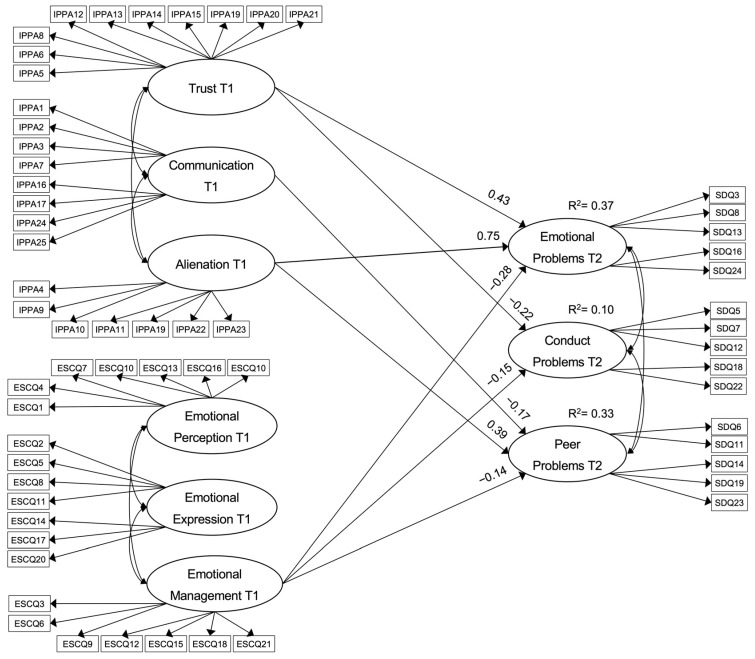
Final model to predict emotional symptoms, conduct problems and peer problems.

In the first estimation of the model, the goodness-of-fit results were fairly satisfactory. That is, the chi-square test was statistically significant, and the other indices showed that the model fit the data well: χ^2^ (1674) = 2936.243, *p* < 0.001, RMSEA = 0.03 (90% CI 0.02–0.04), CFI = 0.91, SRMR = 0.05. However, a number of hypothesised relationships were not statistically significant and therefore could not be considered in the population. These non-significant relationships were then set to zero, and the model was re-estimated. The modified model fit the data equally well and was even more robust (χ^2^ (1684) = 2942.805, *p* < 0.001, RMSEA = 0.03 (90% CI 0.02–0.04), CFI = 0.91, SRMR = 0.04). All factor loadings for latent variables were satisfactory (>0.40) and significant (*p* < 0.001), indicating a good fit to the data from the study sample. The standardised parameter estimators are presented in Figure 2.

First, peer trust had a negative direct effect on conduct problems (β = −0.22; *p* < 0.001), as well as a positive direct effect on emotional symptoms (β = 0.43; *p* < 0.001). Peer communication had only a negative direct effect on peer relationship problems (β = −0.17; *p* < 0.001), while peer alienation showed positive direct effects on emotional symptoms (β = 0.75; *p* < 0.001) and peer relationship problems (β = 0.39; *p* < 0.001). On the other hand, emotional management directly affected conduct problems (β = −0.15; *p* < 0.001), emotional symptoms (β = −0.28; *p* < 0.001) and peer relationship problems (β = −0.14; *p* < 0.001).

The predictive power of the model for each of the dependent variables was estimated through the corresponding R^2^. Thus, the combined direct effects predicted a significant and moderate proportion of the variance of emotional symptoms (37%) and peer relationship problems (33%), but a rather a small proportion of the variance of conduct problems (10%).

### 3.3. Fuzzy Set Qualitative Comparative Analysis (fsQCA)

The descriptive statistics and the calibration values of emotional symptoms, conduct problems, peer problems, peer attachment and emotional competence were calculated (Table 2). Necessary and sufficient conditions were estimated. In terms of necessary conditions, peer attachment and emotional competence were not conditions that must always be present for the high or low levels of emotional symptoms, conduct problems and peer problems to occur (all consistency values were <0.90). As for the sufficient conditions, the results showed different combinations for high and low levels of adjustment difficulties. First, the results obtained for high levels of the variables (emotional symptoms, conduct problems and peer problems) are presented.

In emotional symptoms, the intermediate solution proved to have adequate consistency (overall solution consistency = 0.77) and the combination of causal conditions explained 41% of the cases of high levels of emotional symptoms. There were a total of eight pathways for high levels of emotional symptoms, and the three main combinations are shown in Table 3. Pathway 1 was composed of high levels of trust and alienation, and low levels of emotional management. This first combination explained 28% of the cases of high levels of emotional symptoms. Pathway 2 was composed of high levels of communication and alienation, and low levels of emotional expression and emotional management. That combination explained 26% of the cases of high levels of emotional symptoms. The third pathway was the result of high levels of communication, alienation and emotional perception, and low levels of emotional management, and this combination explained 23% of high emotional symptoms.

For conduct problems, the intermediate solution showed adequate consistency (overall solution consistency = 0.83) and the combination of causal conditions explained 24% of the cases of high levels of conduct problems. Table 3 shows the three pathways obtained for high levels of conduct problems. The was the result of the interaction of low trust and emotional perception, and high communication, alienation and emotional expression, and the pathway explained 21% of the cases of high conduct problems. The second combination was that of low trust and emotional management, and high communication, alienation and emotional expression, and the pathway explained 21% of high conduct problems. The third combination was the result of the interaction of low trust and emotional expression, and high communication, alienation, emotional perception and emotional management. The third combination explained 16% of the cases of conduct problems.

In peer problems, the intermediate solution proved to have adequate consistency (overall solution consistency = 0.80) and it explained 51% of the cases of high levels of peer problems. The number of pathways for high levels of peer problems totalled ten and the three main combinations are shown in Table 3. Pathway 1 was composed of high levels of alienation and emotional perception, and low levels of trust. This combination explained 32% of the cases of peer problems. Pathway 2 consisted of high levels of alienation and emotional expression, and low levels of trust, and the combination explained 30% of peer problems. Pathway 3 was the result of high levels of alienation and emotional perception, and low levels of emotional management. The combination explained 30% of the cases of high peer problems.

Secondly, the main combinations for predicting low levels of the variables (emotional symptoms, conduct problems and peer problems) are provided. For emotional symptoms, the intermediate solution showed adequate consistency (overall solution consistency = 0.84) and it explained 43% of the cases of low levels of emotional symptoms. There were six total pathways for low levels of emotional symptoms, and the three main combinations are shown in Table 4. Combination 1 was composed of low levels of communication and alienation, and high levels of trust and emotional management. That pathway explained 27% of the cases of low emotional symptoms. Combination 2 was composed of low levels of trust and alienation, and high levels of emotional expression and emotional management, and this interaction explained 26% of high levels of emotional symptoms. Combination 3 was the result of high communication and emotional management, and low alienation and perceiving emotions. The third pathway explained 25% of the cases of low emotional symptoms.

For conduct problems, the intermediate solution showed adequate consistency (overall solution consistency = 0.87) and the combination of causal conditions explained 37% of the cases of low levels of conduct problems. The pathways for low levels of problem behaviour totalled eight and the three main combinations are shown in Table 4. Pathway 1 was composed of high levels of confidence, emotional expression and emotional management, and low levels of emotional perception. This combination explained 22% of the cases of low behavioural problems. Pathway 2 was composed of high levels of confidence and emotional management, and low levels of communication and emotional perception, and the combination explained 22% of the cases of conduct problems. Pathway 3 was the combination of high levels of confidence, emotional expression and emotional management, and low levels of alienation and emotional perception. This last pathway explained 20% of the cases of low behavioural problems.

For peer problems, the intermediate solution proved to have adequate consistency (overall solution consistency = 0.75) and the combination of causal conditions explained 65% of the cases of low levels of peer problems. There were ten total pathways for low levels of peer problems, and the three main combinations are shown in Table 4. Pathway 1 was composed of high levels of trust and low levels of emotional perception, and it explained 43% of the low levels of peer problems. Pathway 2 was composed of high trust and low emotional management, and the combination explained 42% of the cases of low peer problems. Pathway 3 was composed of high levels of trust and low levels of communication, and the combination explained 38% of the cases of low peer problems.

## 4. Discussion

The present study examined how peer attachment and emotional competencies impact psychological adjustment in adolescence. During this period of development, adolescents experience significant changes, which makes it important to identify the protective factors that facilitate a positive development and enable good adjustment to the environment. This study aimed to better understand some of the underlying factors that cause some adolescents to struggle with emotional symptoms, behavioural problems and peer problems, while others strive to develop their strengths.

Firstly, the expected outcome was that secure peer attachment (high trust, high communication and low alienation) and optimal emotional competence (low emotional perception, high emotional expression and high emotional management) were negatively associated with emotional symptoms. The results from SEM and fsQCA partially confirmed the first hypothesis and indicated a significant impact of peer attachment and emotional competences on emotional symptoms. Both methodologies revealed that the most relevant predictors for emotional symptoms are alienation and emotional management. The structural equation suggested that trust and alienation were positively associated with emotional symptoms. On the other hand, emotional management was negatively related to emotional symptoms. The results obtained with fsQCA suggested that alienation and emotional management were the most relevant variables in the pathways to explain both high and low levels of emotional symptoms. These results suggest that adolescents who experienced feelings of social isolation and detachment towards peers, as well as difficulties in managing their emotions, were likely to develop excessive worry, low confidence and nervousness in new situations. In contrast, adolescents with an adequate sense of attachment to their peers who were also able to manage their emotions had fewer worries, nervousness and fears. These results were in line with previous studies suggesting that adolescents with insecure attachment will experience more emotional difficulties [33,36].

During this stage, peer relationships may determine both present and future psychosocial adjustment and well-being [74,75]. Thus, the bond established with peers may have a differential influence depending on whether it is positive or negative. For example, an appropriate peer relationship might promote adolescents’ life satisfaction, whereas the same peer relationships, if they involve bullying behaviours, will have a negative influence [76]. Furthermore, the way in which emotions are regulated during this stage may also be a determining factor of the emotional symptoms experienced, as suggested by previous studies [3,51]. Adolescence is a complex stage in which numerous changes occur [1] and an adequate ability of managing emotions could prevent the appearance of affective symptomatology [48,51]. Therefore, developing programs in schools that identify adolescents with difficulties in emotional regulation and high peer alienation may contribute to the early detection of emotional symptoms [48,51].

Contrary to expectations, the dimension of peer attachment trust, adolescents’ perception of mutual trust and respect, was positively associated with emotional problems, especially in SEM models. A tentative explanation could be given by the combination of variables (fsQCA models). In the main pathways, adolescents who had emotional problems and trusted peers were also those who felt isolated from the group and were less able to regulate their emotions. It is possible that relying a lot on peers may be negative if it is not accompanied by feelings of belonging to the group and adequate emotion regulation. This approach is consistent with previous studies showing the importance of the quality of peer relationships and the influence of emotional management during adolescence [53,76].

According to the second hypothesis, secure peer attachment (high trust, high communication and low alienation) and optimal emotional competence (low emotional perception, high emotional expression and high managing emotions) were negatively associated with conduct problems. The results of our study partially support this hypothesis, as they suggest that peer attachment and emotional abilities have a significant effect on conduct problems. The structural equation model indicated that trust and emotional management were the primary predictors of conduct problems, however the effect size was rather small given the large sample. Thus, adolescents who perceive mutual trust and respect in their peer relationships and are able to effectively manage their own emotions are to some extent less likely to develop conduct problems over time.

The results from fsQCA indicated a more diverse combination of predictors, including all dimensions of peer attachment and emotional competences. Adolescents with high levels of conduct problems are more likely to have low trust in others, as well as high alienation and communication. It is striking that high communication is related to high conduct problems, when previous studies suggest the opposite [34,35]. This inconsistency could be explained by the combination between variables. In the main pathways, adolescents with high communication have shown difficulties in certain dimensions of emotional competence. Some adolescents did not express their emotions adequately and those who expressed them correctly had difficulties in regulating or perceiving them. In the social domain, emotions have an essential role to play in social interactions and especially the ability to manage one’s emotions becomes an important skill for initiating and maintaining peer relationships [54]. In this sense, emotional competencies influence peer relationships [55] and adolescents with higher emotional skills may maintain more positive and healthy social interactions [56,58]. In line with suggestions from previous studies [47,48], these results reveal the importance of developing emotional competence during adolescence to prevent the emergence of conduct problems.

FsQCA models also suggested that attachment difficulties might be more relevant as risk factors, while the development of emotional competence may play a role as a protective factor. In other words, when adolescents have low trust in their peers, have inadequate communication and are alienated, they are more likely to have behavioural problems. These results are quite consistent with previous research that shows that the relationship established with peers might influence the emergence of adjustment difficulties [5]. On the contrary, adolescents with adequate emotional perception (low levels) and optimal emotional management are likely to have fewer conduct problems. The results of this study, congruent with previous research conducted with adolescents, showed the protective role of emotional skills in psychological adjustment during this stage [45].

The main differences between the results obtained in the SEM models and the fsQCA models were observed in communication, alienation and emotional perception. In the SEM models, these did not seem to be key variables, whereas in the fsQCA models they appeared to be reflected in the main combinations. Specifically, communication and alienation were found to be relevant in predicting high levels of conduct problems, but not low levels. In contrast, perception was relevant in predicting low levels, but not high levels. In the SEM models, none of the three variables (communication, alienation and perception) were significant in predicting conduct problems. These a priori contradictory results could be understood considering the methodology. In fsQCA models, the variables that predict high levels are not necessarily the same as those that influence the occurrence of low levels [72]. This potential independence between high and low levels (which is not found in SEM models) could be the basis for the differences found. It is possible that variables predicting only high levels or only low levels are not captured in structural equation models. These results show the usefulness of complementing the SEM models with the use of the fsQCA methodology that allows analysing the combined contribution of the variables, as suggested by previous studies [69].

The third hypothesis proposed that secure peer attachment (high trust, high communication and low alienation) and optimal emotional competence (low emotional perception, high emotional expression and high emotional management) were negatively associated with peer problems. The results obtained by both methodologies partially confirmed this hypothesis, showing that peer problems were significantly influenced by peer attachment and emotional competencies. The SEM and fsQCA models both emphasised the prediction of peer problems through trust and alienation.

The results obtained by the structural prediction model indicated that high levels of trust, and to a greater extent low levels of alienation, were negatively related to peer problems. In addition, high levels of emotional management predicted lower levels of peer problems. On the contrary, in the fsQCA model, communication and emotional management appeared in only one of the three main combinations for predicting low levels of peer problems. Hence, they could not be considered as the main conditions influencing the occurrence of peer relationship problems. These results may be supported by the SEM model. In equation models, communication and management were the predictors with the lowest estimators. Overall, the results suggested that alienation was the most relevant variable in the prediction of peer problems. These results are consistent with the literature, which shows that adolescents who isolate themselves from their peer group have more adjustment difficulties [32,33].

In summary, the results obtained from two different methodologies (SEM and fsQCA) indicated that peer attachment and emotional competencies influence adolescent psychological adjustment. On the one hand, adolescents with high peer alienation and low emotional management tend to internalise more emotional symptoms. Furthermore, adolescents with low peer trust and a low ability of managing emotions tend to externalise more conduct problems. On the other hand, adolescents with high peer alienation are more likely to struggle with peer problems.

### Strengths and Limitations

This study provided empirical evidence on the positive influence of peer attachment and emotional competencies on adolescent adjustment based on a solid methodological design and longitudinal data. The combination of two different methodologies in the prediction of adolescent adjustment has important advantages. On the one hand, SEM is a multivariate technique that allows estimating latent variables (constructs) from observed variables (items) and measurement error, testing whether a theoretical or hypothesised model fits the sample data. On the other hand, fsQCA models are exploratory rather than confirmatory, offering a variety of pathways where predictors can be combined in different ways, also including a greater multiplicity of factors than SEM models. FsQCA models are therefore a statistical analysis technique that could complement SEM models.

Despite the strengths of this study, some limitations should be noted. First, the sampling method used in this study was non-probabilistic, which means that there may be a sample selection bias, limiting the inferences that can be made about the population. In future studies, researchers should consider using a stratified random cluster sampling that includes adolescents from different socio-economic and cultural backgrounds, representing the entire population. Second, data have been collected exclusively via self-reports. Although adolescents are considered capable of reporting honestly about their personal thoughts, feelings and behaviours, these self-reports are inherently subjective and may be biased. Therefore, it would be advisable to also include performance measures of emotional skills (MESCEIT [77]) and multiple sources of information from parents, teachers and peers in future research. Third, the data could also be compromised by random, pseudorandom, or dishonest responses. Infrequency scales should be incorporated in future research to ensure the reliability of the data collected.

## 5. Conclusions

The limitations of this study notwithstanding, this research contributes to the exploration of potential risk and protective factors for psychological adjustment in adolescence. Alienation and trust problems with peers could be considered as the main risk factors for the emergence of psychological adjustment problems in adolescence, while appropriate emotional management seems to be a protective factor. On the one hand, the importance of adolescents’ relationships with their peers was evident, considering their influence on psychological adjustment. In this sense, the need to foster a positive climate in the schools and to strengthen the bonds between adolescents was confirmed. On the other hand, the influence of emotional development in this complex stage was undeniable. These findings contribute to highlighting the relevance of including emotional education programmes that foster positive relationships among students and promote appropriate emotional management.

## Figures and Tables

**Table 1:** Descriptive statistics and Person’s correlations between variables.

Variable	Range	*M*	*SD*	1	2	3	4	5	6	7	8
Peer Attachment											
1. Trust T1	11–50	41.84	6.29	-							
2. Communication T1	11–40	30.16	5.81	0.70 **	-						
3. Alienation T1	6–30	16.36	4.06	−0.44 **	−0.23 **	-					
Emotional competence											
4. Emotional perception T1	27–90	66.19	10.03	0.40 **	0.47 **	−0.19 **	-				
5. Emotional expression T1	22–84	63.10	9.50	0.40 **	0.47 **	−0.15 **	0.85 **	-			
6. Emotional management T1	22–96	70.50	10.53	0.42 **	0.48 **	−0.18 **	0.85 **	0.84 **	-		
Adjustment											
7. Emotional symptoms T2	5–15	8.08	2.23	−0.20 **	−0.09 *	0.37 **	−0.21 **	−0.16 **	−0.20 **	-	
8. Conduct problems T2	4–15	6.24	1.79	−0.17 **	−0.12 **	0.15 **	−0.08 *	−0.13 **	−0.13 **	0.29 **	-
9. Peer problems T2	3–15	6.28	1.87	−0.21 **	−0.14 **	0.22 **	−0.05	−0.05	−0.06	0.26 **	0.29 **

Note: * *p* < 0.05. ** *p* < 0.01.

**Table 2:** Descriptive statics and calibration values.

Descriptive Statistics		Emot. Symp.	Cond. Prob.	Peer Prob.	Trust	Comm.	Alien.	Percep.	Express.	Manag.
Mean		16.49	8.15	6.01	29,378.04	82,593.70	185.49	60,535.30	50,869.28	62,481.10
Standard deviation		29.65	15.80	9.89	28,131.39	96,048.90	456.20	64,004.63	66,117.02	65,885.48
Minimum		1.00	1.00	1.00	0.48	8.00	1.00	2.00	1.00	1.00
Maximum		243.00	162.00	81.00	97,656.25	390,625.00	5000.00	279,936.00	279,936.00	279,936.00
Calibration values										
Percentile	10	1.00	1.00	1.00	1573.12	2707.20	4.00	5760.00	720.00	4320.00
	50	6.00	4.00	2.00	19,531.25	46,080.00	48.00	40,000.00	24,000.00	40,000.00
	90	36.00	17.20	16.00	78,125.00	220,625.00	400.00	155,520.00	135,000.00	162,000.00

Note: Emot. symp. = Emotional symptoms; Cond. prob. = Conduct problems; Peer prob. = Peer problems; Comm. = Communication; Alien. = Alienation; Percep. = Emotional perception; Express. = Emotional expression; Manag. = Emotional management.

**Table 3:** Combinations from intermediate solutions for high levels of emotional symptoms, conduct problems and peer problems.

Conditions	High Level of Emotional Symptoms (T2)			High Level of Conduct Problems (T2)			High Level of Peer Problems (T2)		
	1	2	3	1	2	3	1	2	3
Trust (T1)	●			○	○	○	○	○	
Communication (T1)		●	●	●	●	●			
Alienation (T1)	●	●	●	●	●	●	●	●	●
Emotional perception (T1)			●	○		●	●		●
Emotional expression (T1)		○		●	●	○		●	
Emotional management (T1)	○	○	○		○	●			○
Consistency	0.82	0.84	0.84	0.85	0.85	0.85	0.84	0.82	0.84
Raw Coverage	0.28	0.26	0.23	0.21	0.21	0.16	0.32	0.30	0.30
Unique Coverage	0.018	0.025	0.002	0.009	0.010	0.014	<0.001	0.001	<0.001
Overall Solution Consistency			0.41			0.24			0.51
Overall Solution Coverage			0.77			0.83			0.80

Note: ● = Presence or high levels. ○ = Absence or low levels. All paths are consistent because consistency is above 0.74.

**Table 4:** Combinations from intermediate solutions for low levels of emotional symptoms, conduct problems and peer problems.

Conditions	Low Level of Emotional Symptoms (T2)			Low Level of Conduct Problems (T2)			Low Level of Peer Problems (T2)		
	1	2	3	1	2	3	1	2	3
Trust (T1)	●	○		●	●	●	●	●	●
Communication (T1)	○		●		○				○
Alienation (T1)	○	○	○			○			
Emotional perception (T1)			○	○	○	○	○		
Emotional expression (T1)		●		●		●			
Emotional management (T1)	●	●	●	●	●	●		○	
Consistency	0.88	0.88	0.89	0.90	0.89	0.90	0.83	0.79	0.85
Raw Coverage	0.27	0.26	0.25	0.22	0.22	0.20	0.43	0.42	0.38
Unique Coverage	0.027	0.052	0.014	<0.001	0.027	0.017	0.005	0.001	0.021
Overall Solution Consistency			0.43			0.37			0.65
Overall Solution Coverage			0.84			0.87			0.75

Note: ● = Presence or high levels. ○ = Absence or low levels. All paths are consistent because consistency is above 0.74.

## Data Availability

The datasets generated and/or analysed during the current study are available from the corresponding author upon reasonable request.
